# Disparities in mammographic screening for Asian women in California: a cross-sectional analysis to identify meaningful groups for targeted intervention

**DOI:** 10.1186/1471-2407-7-201

**Published:** 2007-10-26

**Authors:** Scarlett Lin Gomez, Susanna Tan, Theresa HM Keegan, Christina A Clarke

**Affiliations:** 1 Northern California Cancer Center, 2201 Walnut Ave, Suite 300, Fremont, CA, 94538, USA; 2 Department of Health Research and Policy, Stanford University School of Medicine, Stanford, CA, USA; 3 Northwestern Feinberg School of Medicine, Northwestern University, Chicago, IL, 94305, USA

## Abstract

**Background:**

Breast cancer is the most commonly diagnosed cancer among the rapidly growing population of Asian Americans; it is also the most common cause of cancer mortality among Filipinas. Asian women continue to have lower rates of mammographic screening than women of most other racial/ethnic groups. While prior studies have described the effects of sociodemographic and other characteristics of women on non-adherence to screening guidelines, they have not identified the distinct segments of the population who remain at highest risk of not being screened.

**Methods:**

To better describe characteristics of Asian women associated with not having a mammogram in the last two years, we applied recursive partitioning to population-based data (N = 1521) from the 2001 California Health Interview Survey (CHIS), for seven racial/ethnic groups of interest: Chinese, Japanese, Filipino, Korean, South Asian, Vietnamese, and all Asians combined.

**Results:**

We identified two major subgroups of Asian women who reported not having a mammogram in the past two years and therefore, did not follow mammography screening recommendations: 1) women who have never had a pap exam to screen for cervical cancer (68% had no mammogram), and 2) women who have had a pap exam, but have no women's health issues (osteoporosis, using menopausal hormone therapies, and/or hysterectomy) nor a usual source of care (62% had no mammogram). Only 19% of Asian women who have had pap screening and have women's health issues did not have a mammogram in the past two years. In virtually all ethnic subgroups, having had pap or colorectal screening were the strongest delineators of mammography usage. Other characteristics of women least likely to have had a mammogram included: Chinese non-U.S. citizens or citizens without usual source of health care, Filipinas with no health insurance, Koreans without women's health issues and public or no health insurance, South Asians less than age 50 who were unemployed or non-citizens, and Vietnamese women who were never married.

**Conclusion:**

We identified distinct subgroups of Asian women at highest risk of not adhering to mammography screening guidelines; these data can inform outreach efforts aimed at reducing the disparity in mammography screening among Asian women.

## Background

Asians and Pacific Islanders are among the most rapidly growing racial/ethnic population groups in the United States (U.S.), with most of the growth attributable to high immigration rates from Asian and Pacific Island countries [1,2]. California is one of the main geographic targets of this immigration, such that more than one-third of all U.S. Asians and Pacific Islanders (API), or four million people, now live in California [3-7]. Growth in API groups is expected to continue over time, with a projected population of 11 million California APIs by 2025 [7].

Breast cancer consistently is the most commonly diagnosed cancer and the second most common cause of cancer mortality among U.S. Asians; among Filipinas, it is the most common cause of cancer mortality [8,9]. Despite overwhelming clinical evidence supporting the efficacy of mammograms for reducing breast cancer mortality [10] and the Healthy People 2010 objective to have 70% of U.S. women age 40 and older receive a mammogram at least once every two years, recent population risk factor surveys have shown that use of mammography continues to vary by racial/ethnic group [11-13]. According to the 2001 California Health Interview Survey (CHIS), Whites, African Americans, and Latinas have already met the national objective of having a mammogram in the past two years (78.1%, 78.5%, and 69.9%, respectively) but Asians (67.2%) and Native Hawaiians/other Pacific Islanders (63.4%) lag behind [13]. Like other women, Asian Americans may not follow mammography screening guidelines because of practical considerations such as lack of time, money, health insurance, transportation, or having a usual source of care [14-25]; lack of encouragement by physicians or family [14,16,22,24-27]; and perceptions that mammograms are inconvenient, uncomfortable, or dangerous; or perceptions that breast cancer is not a serious illness [24,26,28]. However, sociocultural factors more relevant to immigrant groups especially those recently immigrated to the U.S., like low level of education [18,26], inability to speak English [19,25,29,30], low level of acculturation [17,18,22,24,26,27,31], and racial/ethnic or cultural discordance with providers [17,32], are also associated with low mammography utilization.

While prior studies have been able to identify characteristics associated independently with use of mammography screening, most of these studies have been conducted with convenience-based, rather than population-based, representative samples of women. Moreover, relying on standard regression techniques, nearly all previous studies have identified independent effects of socioeconomic and other characteristics associated with mammography use. With these traditional techniques, it is more difficult to consider the complex interactions among multiple factors for describing important high risk groups most likely not to comply with mammography screening recommendations [33]. Public health interventions would be most effective and cost efficient if applied to segments of the population most at risk. Thus, in this analysis, we applied the novel statistical method recursive partitioning (RP) to a large, population-based resource to identify clustered characteristics of Asian American women most likely not to follow mammography screening guidelines and who might benefit from targeted intervention. We conducted analyses separately for Asians as a group as well as for ethnic subgroups, because of demonstrated heterogeneity across these subgroups in screening rates [34,35] and the proportion of early stage diagnoses (51%, 41%, and 35% stage I breast cancer among Japanese, Chinese, and Filipinas, respectively [36]).

## Methods

### CHIS data and study sample

We used population-based data from the 2001 CHIS, a telephone survey based on a geographically stratified sample, identified through random-digit-dialing (RDD) that over-sampled under-represented geographic areas and ethnic groups. The data collected by CHIS, the largest state health survey, are intended to provide health planners, policy makers, county governments, advocacy groups, and communities a detailed picture of the health and health care needs among California's diverse population [37]. The CHIS sample represents the geographic and racial/ethnic diversity of California; interviews are conducted in multiple languages to accommodate the state's rich racial/ethnic diversity. CHIS 2001 collected information from more than 55,000 households across California and conducted interviews in seven languages: English, Spanish, Mandarin, Cantonese, Vietnamese, Korean, and Cambodian. Among the households who completed a screening interview (59.2% of RDD sample), the response rate for the adult interview across the state was 63.7% [38] for an overall response rate of 37.7%, which was comparable to response rates among Asians: South Asian (39.5%), Japanese (35.3%), Korean (42.5%), and Vietnamese (35.3%); response rates for Chinese and Filipino were not available from CHIS as they were included in the larger RDD samples and not in the ethnicity-specific oversampling efforts [39].

This analysis included women of self-reported (or CHIS-imputed) Asian ethnicities who at the time of interview, did not report a prior history of breast cancer and who were aged 41 years or older. This age cut-off for sample selection was chosen because of screening guidelines recommending annual mammograms starting at age 40 [40]. Those with prior history of breast cancer were excluded (N = 39 women who reported a prior diagnosis of breast cancer, and N = 5 women for whom this status was not ascertained) because their screening habits are likely influenced by their prior diagnoses and routine medical surveillance. We excluded 22 women for whom mammography status was not ascertained. Thus, our final analytic sample (N = 1521) included women with the following ethnic identifications: Chinese (n = 382), Japanese (n = 275), Filipino (n = 269), Korean (n = 244), South Asian (n = 125), and Vietnamese (n = 226). The South Asian subgroup includes women of self-reported descent from South Asian countries such as India, and Pakistan.

### Outcome and explanatory variables for recursive partitioning

RP [41,42] was used to identify mutually exclusive subgroups, with variations in outcome, delineated by combinations of explanatory characteristics. The dichotomous outcome variable represented categorization of responses to the question "How long ago did you have your most recent mammogram?". We selected a cut-off of two years in reference to the Healthy People 2010 goal of having 70% of women in the United States receive a mammogram within the past two years [43]. In addition, women were considered to have received a screening mammogram within the past two years if it was conducted "as part of a routine physical exam or screening"; other reasons (7% of respondents), including "because of a specific breast problem", "as a follow-up to a previously identified breast problem", and "as a result of a baseline or initial mammogram" were considered mammograms that were conducted for diagnostic purposes.

The 25 explanatory variables submitted into the RP procedure are presented in Table 1. These variables were selected from among the CHIS questions because they have previously been shown or were hypothesized to be associated with mammography screening. Two of us (SLG & ST) independently reviewed the CHIS variables and selected those to be included in the RP analysis; discrepancies were resolved by discussion. Prior to RP analysis, several variables were modified from their original CHIS format, including income, which was adjusted for household size, and percentage of lifetime in the U.S., which was derived by dividing the number of years lived in the U.S. by age in years. Categories of English-speaking proficiency (speaks English only, very well, well, or not well) and education were grouped (Table 1) according to the distributions for each subgroup to overcome sparse numbers in the sub-categories. We also included a variable designating eligibility for public programs, including public housing subsidies, general assistance and relief, food stamps, disability, or Women, Infants, and Children (WIC). We included two variables characterizing pre-existing health conditions: 1) having had pre-existing health issues, which included self-report of at least one of the following: arthritis, asthma, diabetes, or high blood pressure; 2) having women's health issues, which included at least one of the following: taking hormone supplements, history of osteoporosis, or hysterectomy. We created a variable indicating ever having had a Pap exam to screen for cervical cancer based on the question "Have you ever had a Pap smear test to check for cervical cancer." We created another variable indicating ever having been screened for colorectal cancer based on the questions "Have you ever had a Sigmoidoscopy, Colonoscopy, or a Proctoscopy to look for signs of cancer or other problems in your colon" and "Have you ever done a blood stool test, using a home test kit?"; women were considered to have ever been screened for colorectal cancer if their most recent colorectal exam was conducted "as part of a routine physical exam or screening test"; other reasons including "because of a specific problem" or "as a follow-up to an earlier test or screening exam" were considered procedures done for diagnostic purposes. We also combined the Pap and colorectal cancer screening variables into one variable indicating ever use of other cancer screening tests. For all of the explanatory variables, "refused", "don't know", or "not ascertained" responses to survey questions were coded as missing in the analysis.

**Table 1:** Distribution of Asian women according to mammogram (mam) status, by selected characteristics^1^

**Characteristic**	**N (row %) Mam in past 2 yrs, N = 981**	**N (row %) No mam in past 2 yrs, N = 540**	**Total (N = 1521)**
Asian subgroup			
Chinese	246 (64)	136 (36)	382
Japanese	194 (71)	81 (29)	275
Filipina	178 (66)	91 (34)	269
Korean	127 (52)	117 (48)	244
South Asian	79 (63)	46 (37)	125
Vietnamese	157 (69)	69 (31)	226
	p-value^2 ^=< .0001		
Age at interview			
41–49	353 (59)	248 (41)	601
50–64	391 (70)	164 (30)	555
65+	237 (65)	128 (35)	365
	p-value = .0002		
English speaking proficiency (Chinese, Korean, Vietnamese)	530	322	
English not well	294 (59)	204 (41)	498
English well, very well, only	236 (67)	118 (33)	354
	p-value = .024		
English speaking proficiency (Filipino, Japanese, South Asian)	451	218	
English well, not well	146 (65)	77 (35)	223
English very well, only	305 (68)	141 (32)	446
	p-value = .448		
Education (excluding Vietnamese)	824	471	
High school diploma or less	248 (62)	155 (38)	403
Some college, vocational school	187 (63)	109 (37)	296
Bachelor's degree or more	389 (65)	207 (35)	596
	p-value = .477		
Education (only Vietnamese)	157	69	
High school diploma or less	123 (69)	56 (31)	179
Some college or more	34 (72)	13 (28)	47
	p-value = .631		
Annual household income adjusted for household size			
<$10,000 (per household member)	186 (60)	123 (40)	309
$10,000–$19,999	269 (61)	173 (39)	442
$20,000–$33,749	261 (69)	118 (31)	379
$33,750+	265 (68)	136 (32)	391
	p-value = .018		
Employer type	968	531	
Unemployed	458 (66)	241 (34)	699
Private	325 (63)	190 (37)	515
Federal, state, local	114 (71)	47 (29)	161
Self-employed, family business	71 (57)	53 (43)	124
	p-value = .094		
Employment hours (per week)			
Unemployed	458 (65)	243 (35)	701
1–30 hours	112 (62)	69 (38)	181
>30 hours	411 (64)	228 (36)	639
	p-value = .682		
Health insurance			
No	76 (44)	97 (56)	173
Yes	905 (67)	443 (33)	1348
	p-value =< .0001		
Type of health insurance (among insured)	905	443	
Public (Medicaid only, Medicare only, Medicare & Medicaid, other public)	192 (60)	127 (40)	319
Private (employment-based, private, Medicare & private)	713 (69)	316 (31)	1029
	p-value = .003		
Managed care health plan (among insured)	877	427	
No	291 (65)	160 (35)	451
Yes	586 (69)	267 (31)	853
	p-value = .127		
Public program eligibility (public housing subsidies, general assistance and relief, food stamps, disability, WIC)			
No	841 (65)	456 (35)	1297
Yes	140 (63)	84 (37)	224
	p-value = .499		
Marital status	979	540	
Married	620 (65)	331 (35)	951
Widowed, divorced, separate, living with partner	290 (64)	160 (36)	450
Never married	69 (58)	49 (42)	118
	p-value = .355		
Percent of lifetime lived in U.S.			
<10% of life in U.S.	32 (40)	49 (60)	81
10–25% of life in U.S.	138 (61)	89 (39)	227
>25% of life in U.S.	580 (65)	309 (35)	889
U.S. born	231 (71)	93 (29)	324
	p-value < .0001		
Citizenship status			
U.S. born	231 (71)	93 (29)	324
Naturalized citizen	600 (68)	283 (32)	883
Non-citizen	150 (48)	164 (52)	314
	p-value < .0001		
Usual source of care	980	539	
No	56 (38)	90 (62)	146
Yes	924 (67)	449 (33)	1373
	p-value < .0001		
Went to another country for care			
No	959 (65)	526 (35)	1485
Yes	22 (61)	14 (39)	36
	p-value = .667		
Personal history of (non-breast) cancer	979	539	
No	944 (64)	524 (36)	1468
Yes	35 (70)	15 (30)	50
	p-value = .408		
Blood relative with cancer	974	535	
No	633 (62)	380 (38)	1013
Yes	341 (69)	155 (31)	496
	p-value = .017		
Blood relative with breast cancer			
No	943 (64)	526 (36)	1469
Yes	38 (73)	14 (27)	52
	p-value = .188		
Pre-existing health condition (arthritis, asthma, diabetes, high blood pressure)			
No	466 (62)	287 (38)	753
Yes	515 (67)	253 (33)	768
	p-value = .035		
Use of other screenings (pap exam and/or colon-rectal exam)			
No	46 (29)	113 (71)	159
Yes	935 (69)	427 (31)	1362
	p-value < .0001		
Ever had pap smear to check for cervical cancer			
No	62 (32)	131 (68)	193
Yes	919 (69)	409 (31)	1328
	p-value < .0001		
Ever had colon-rectal screening exam			
No	589 (58)	432 (42)	1021
Yes	392 (78)	108 (22)	500
	p-value < .0001		
Has women's health issues (has osteoporosis, takes hormone supplements to control menopause, had hysterectomy)			
No	568 (57)	426 (43)	994
Yes	413 (78)	114 (22)	527
	p-value < .0001		

^1 ^Distribution* of Asian women according to mammogram status (mammogram within the past two years), by selected characteristics, CHIS 2001 (N = 1521); relative distributions are not adjusted for CHIS sampling weights.

^2 ^p-value for chi-square test comparing the distribution among non-users to users.

### Statistical analysis & recursive partitioning

We used RP, a non-parametric method that produces a classification tree in which subjects are assigned to mutually exclusive subsets according to a set of explanatory variables. Unlike traditional regression methods, highly correlated variables can be entered simultaneously because RP manages variables individually. The first step in RP involves examining each explanatory variable and selecting one binary split across the sample on one explanatory variable that minimizes the within-group variance in the outcome variable in the two resultant nodes. This process is repeated for subsequent explanatory variables until further partitioning is not possible; however, the final tree, with small numbers of subjects in the terminal nodes (minimum of 5, per our stopping rule), is subject to high misclassification errors. Therefore, the next step involves "pruning" the tree by sequentially cutting away terminal nodes. The optimal tree, selected via cross-validation (ten-fold in our analysis), has the most splits but the lowest misclassification rate [44]. Additional details about RP are available elsewhere [42,44-46]. RP analyses were conducted using the RPART routine in the R statistical software program [45]. For the variables English proficiency and education, for which two versions of each variable are created depending on the Asian subgroup, the values are set to "missing" for individuals for whom the variable does not apply. For example, for the variable education (among only Vietnamese), this variable is set to missing for women who are not Vietnamese. One of the most powerful advantages of the RPART procedure is its ability to retain observations with partially missing data [45]. Probabilities (and corresponding 95% confidence intervals) of not having had a mammogram in the past two years are computed for each tree terminal node (represented by rectangles); testing for statistically significant differences in these probabilities among groups was not conducted. In the RP analysis, screening for other cancers were the major and sometimes only identified delineator for most groups. In the case where screening for other cancers was the only identified delineator, a separate RP analysis was conducted excluding this variable in order to identify other salient characteristics influencing mammography screening that might better inform interventions.

### Human subjects protection

Institutional review board (ethical) approval was not sought for this research as it was based on a de-identified, public-use dataset.

## Results

Table 1 shows the numbers and relative distributions of the 1521 study subjects according to mammography use within the past two years, and sociodemographic factors. Overall, 540 (35.5%) of all Asian women aged 41 or older reported not having a mammogram in the past two years. This proportion ranged from 29% among Japanese to 48% among Koreans and differed most notably by age at interview, presence of health insurance, percent of lifetime lived in the U.S., citizenship status, having a usual source of care, use of other cancer screening tests, and having women's health issues. Smaller differences in the proportion of mammogram users were also seen by English language proficiency (among Chinese, Koreans, and Vietnamese), income, type of health insurance, family history of cancer, and pre-existing health conditions.

**Figure 1:**
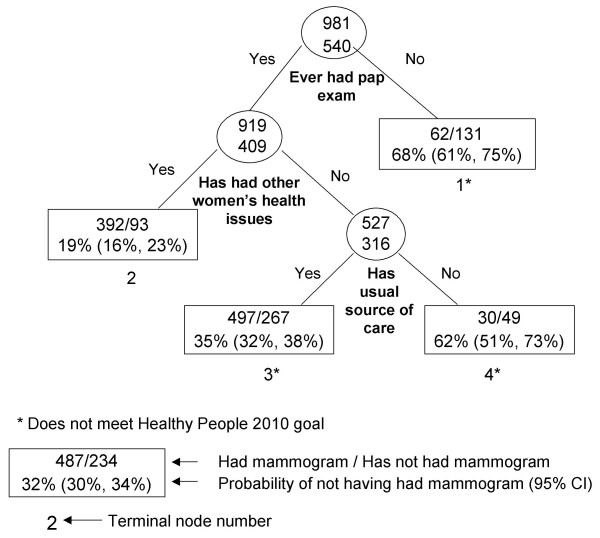
Recursive partitioning classification tree for Asian females 41 years and older, CHIS 2001.

**Figure 2:**
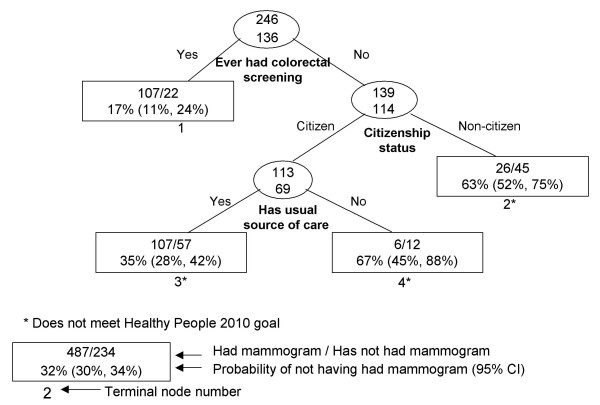
Recursive partitioning classification tree for Chinese females 41 years and older, CHIS 2001.

**Figure 3:**
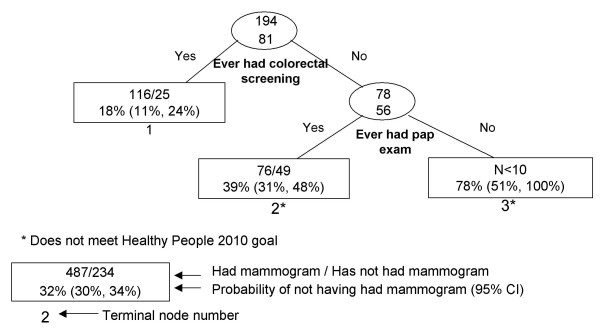
Recursive partitioning classification tree for Japanese females 41 years and older, including pap and colon screening variable, CHIS 2001.

**Figure 4:**
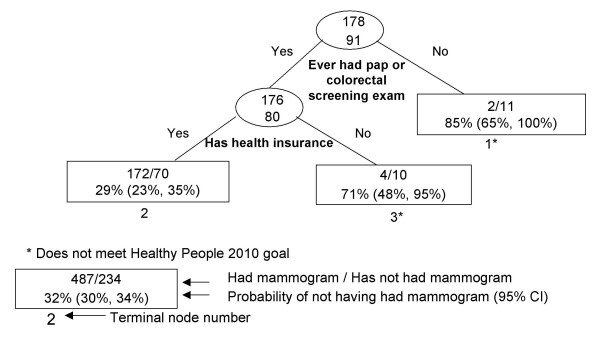
Recursive partitioning classification tree for Filipina females 41 years and older, CHIS 2001.

**Figure 5:**
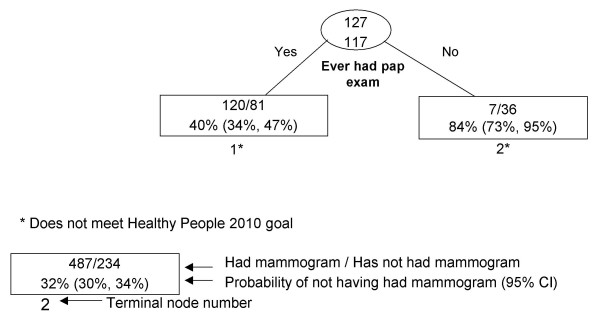
Recursive partitioning classification tree for Korean females 41 years and older, including pap and colon screening variables, CHIS 2001.

**Figure 6:**
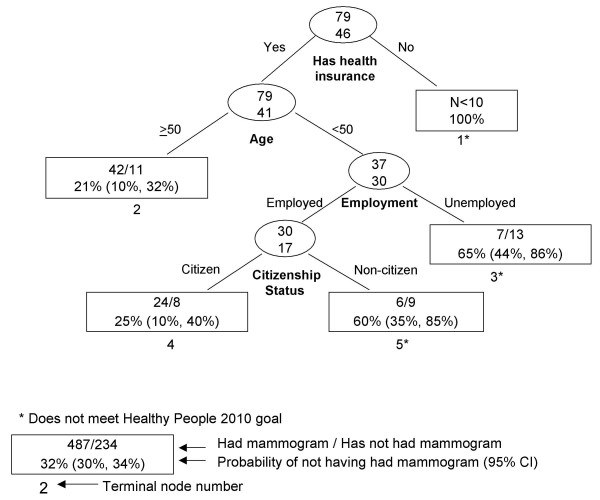
Recursive partitioning classification tree for South Asian females 41 years and older, CHIS 2001.

**Figure 7:**
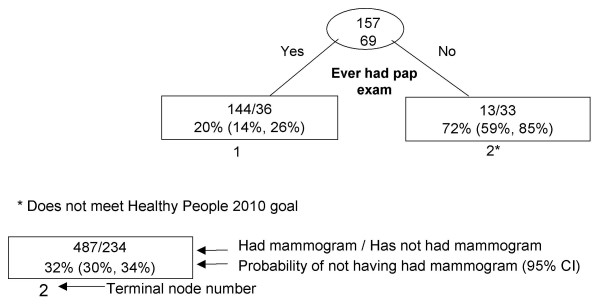
Recursive partitioning classification tree for Vietnamese females 41 years and older, including pap and colon screening variables, CHIS 2001.

RP results are presented graphically (Figures 1, 2, 3, 4, 5, 6, 7) with probabilities of not having a mammogram for relevant subgroups detailed in Table 2. Among all Asian subgroups combined, the optimal RP tree was based on splits of three explanatory variables (Figure 1): 1) ever having a pap screening exam, 2) having other women's health issues, and 3) having a usual source of care. Having a pap exam was the strongest delineator. 68% of Asian women who had not received a pap exam also did not have a mammogram in the past two years (node 1). Another high-risk group (node 4) comprised 62% of women who did not have a mammogram; these were women who have had a pap exam, but have had no other women's health issues and no usual source of care.

**Table 2:** Probability of not having mammogram among risk groups identified from recursive partitioning

**Node**	**Risk group characteristics**	**N**	**Probability of not having a mammogram in the past two years (95% CI)**
All Asians combined (N = 1521)			
1	Never had pap exam	193	68% (61%, 75%) *
2	Ever had pap exam. Has women's health issue(s).	485	19% (16%, 23%)
3	Ever had pap exam. No women's health issues. Has usual source of care.	764	35% (32%, 38%)*
4	Ever had pap exam. No women's health issues. No usual source of care.	79	62% (51%, 73%)*
Chinese (N = 382)			
1	Ever had colorectal cancer screening.	129	17% (11%, 24%)
2	Never had colorectal cancer screening. Non U.S. citizen.	71	63% (52%, 75%)*
3	Never had colorectal cancer screening. U.S. citizen. Has usual source of care.	164	35% (28%, 42%)*
4	Never had colorectal cancer screening. U.S. citizen. No usual source of care.	18	67% (45%, 88%)*
Japanese (N = 275)			
1	Ever had colorectal cancer screening.	141	18% (11%, 24%)
2	Never had colorectal cancer screening. Ever had pap exam.	138	39% (31%, 48%)*
3	Never had colorectal cancer screening. Never had pap exam.	<10	78% (51%, 100%)*
Filipina (N = 269)			
1	Never had pap or colorectal cancer screening.	13	75% (65%, 100%)*
2	Ever had pap or colorectal cancer screening. Has health insurance.	242	29% (23%, 35%)
3	Ever had pap or colorectal cancer screening. Has no health insurance.	15	71% (48%, 95%)*
Korean (N = 244)			
1	Ever had pap exam.	201	40% (34%, 47%)*
2	Never had pap exam.	43	84% (73%, 95%)*
South Asian (N = 125)			
1	Has no health insurance.	<10	100%
2	Has health insurance. Age ≥ 50.	53	21% (10%, 32%)
3	Has health insurance. Age < 50. Unemployed.	20	65% (44%, 86%)*
4	Has health insurance. Age < 50. Employed. U.S. citizen.	32	25% (10%, 40%)
5	Has health insurance. Age < 50. Employed. Non U.S. citizen.	15	60% (35%, 85%)*
Vietnamese (N = 226)			
1	Ever had pap exam.	180	20% (14%, 26%)
2	Never had pap exam.	46	72% (59%, 85%)*

*does not meet HP 2010 goal

In Chinese females (Figure 2), the strongest delineator of mammography screening was having ever had a colorectal screening exam. Among Chinese women who have had a colorectal screening exam, 17% did not have a mammogram within the past two years (node 1), compared to 63% among women who did not have a colorectal exam and were non-U.S. citizens (node 2). Another high-risk group was Chinese women who did not have a colorectal exam, were U.S. citizens, and had no usual source of care (node 4).

Colorectal and pap screening exams were the only two delineating variables in Japanese females (Figure 3). The lowest risk group was Japanese women who have ever been screened for colorectal cancer (node 1), and the highest risk group was Japanese women who have never been screened for colorectal or cervical cancer (node 3). Because screening for other cancers was selected as the only delineator of mammography screening, we conducted RP excluding the pap and colorectal cancer screening variables (data not shown). The lowest risk groups were Japanese women with women's health issues, and women with women's health issues, a usual source of care, and who were unemployed. The resultant high risk groups were based on small numbers of people.

The most discriminating explanatory variable for Filipinas (Figure 4) was ever having any pap or colorectal screening exam; 85% of women without either exam also did not have a mammogram within the past two years (node 1). Among women who have had at least one pap or colorectal exam, having health insurance was an important delineator, as 71% of those without health insurance did not have a mammogram (node 3).

Among Korean females (Figure 5), ever having a pap exam was the most important delineator of mammography use. Among Korean women who have never had a pap exam, 84% have not had a mammogram (node 2). When RP analysis excluded pap and colorectal cancer screening variables (data not shown), women's health issues and health insurance type were found to be additionally important delineators. The lowest risk group was Korean women who have other women's health issues, while the highest risk group was Korean women who did not have other women's health issues and had public or no health insurance.

Among South Asians (Figure 6), health insurance, age, employment, and citizenship status were identified as important delineating variables. The highest risk groups were nodes 1 (women with no health insurance), 3 (women with health insurance, younger than age 50, and were unemployed), and 5 (women with health insurance, younger than age 50, were employed, and were non-citizens).

Among Vietnamese women (Figure 7), 72% of women without a pap exam did not had a mammogram in the previous two years (node 2); however, this was the only discriminating variable selected by RP. Excluding the pap and colorectal cancer screening variables (data not shown), marital status emerged as an additionally important predictor; among Vietnamese women who have never been married, 67% did not have a mammogram in the previous two years. Additional analyses showed that Vietnamese women who have never been married were significantly more likely than women who have been married to have had pap and/or colorectal screening, and were more likely (not significant) to be employed more than 30 hours per week and to receive public assistance.

## Discussion

Despite overall improvements in mammography screening nationwide, there remain disparities in screening among certain population subgroups, including Asians. This study, an analysis of data from a large, population-based sample of Asian women in California, takes advantage of a novel statistical method with the ability to identify discrete subgroups of women who do not appear to be following screening guidelines. This method identified two important characteristics defining Asian women in this category: 1) those who have never had a pap exam to screen for cervical cancer, and 2) those who do not use hormone therapy, have osteoporosis, or have not had a hysterectomy. Previous studies have not reported any of these characteristics as predictors of mammography use. We also found that among women meeting either 1) or 2) above, those with no usual source of health care, were also likely to not follow mammography screening guidelines. Having no usual source of health care has been reported previously as a determinant of mammography use [12,14,24]. Our findings suggest that Asian women who do not follow mammography screening recommendations also do not follow guidelines for other cancer screening tests and may not have good access to health care, so effective programs to improve screening in these groups should focus not just on mammography but on all cancer screening tests. Women's health issues may be a key discriminator of regular recipients of mammograms, as this finding suggests that women may be referred or reminded more often by their physician to obtain mammograms and/or are more diligent about their health in general. In addition, providers treating these conditions may be more likely to be specialists in women's health, and thus more diligent in referring patients to mammography screening. Furthermore, the relationship between using hormone replacement therapy, and possibly treatment for osteoporosis, and breast cancer etiology may result in increased vigilance and surveillance for cancer [42,46-49] thereby prompting providers to recommend routine mammograms for these women. However, it does not appear that routine use of the health care system for other health issues is related to mammography use, as having arthritis, asthma, diabetes, or high blood pressure was not associated with having a mammogram in the past two years in our analysis. Additional focus should be placed on primary care physicians to promote age-appropriate cancer screening. Health plans should also have systems enabling health personnel to issue reminders or even schedule screening appointments for patients who are being seen for other health issues.

The heterogeneity of Asians as a single group [50] is supported by our subgroup-specific findings, and should be emphasized when developing programs targeting specific Asian ethnic communities. Specifically, Chinese non-citizens were at highest risk for not having a mammogram within the past two years. Citizenship status has not previously been shown to be a determinant of mammography use; it may be related to predictors of mammography use found in other studies such as poorer language proficiency, decreased acculturation, and foreign birth [17-19,22,24-27,29-31], although language, education, and birthplace did not emerge as being particularly salient predictors in our study. Being a U.S. citizen may also be associated with having health insurance benefits, familiarity with and trust in Western biomedicine and facility in navigating the American health care system. Among Filipinas who have had pap or colorectal cancer screening, having health insurance was the next strongest delineator of adherence to screening guidelines among Filipinas; this characteristic has been noted elsewhere [27]. Other than pap screening, Korean women without women's health issues and either public or no health insurance had the lowest mammography use, and this finding is supported by previous studies showing lack of insurance as a barrier to mammography screening [51]. In South Asians, we found that despite having health insurance and being employed, non-citizens under age 50 were at high risk of not having a mammogram. Among Vietnamese women, pap screening was an important predictor, as was single marital status, a finding reported previously [17,18,24,26]. Future studies should focus on elucidating explanations of these predictors, and outreach efforts to target these subgroups, particularly among South Asian and Vietnamese women, for whom considerably less research has been conducted.

This study has a number of strengths. We used RP, an emerging but currently under-used, powerful statistical technique to identify meaningful subgroups of Asian women at high-risk for not following mammogram screening recommendations. RP is suitable for identifying high-risk subgroups as it creates combinations of variables that best describe mammography use. Because of the exploratory nature of this technique, we found several factors associated with mammography use that have not previously been shown in the literature. However, RP also confirmed other factors, such as health insurance and marital status, which have previously been shown to be associated with mammography screening among Asians, suggesting that RP can be reliably applied to this research question. This technique was applied recently to a prospective study of 1229 African-American and White women in Connecticut to similarly identify subgroups of women who did not adhere to mammogram screening guidelines [33]. Our study, however, is the first, to our knowledge to apply RP to a large population-based behavioral risk factor survey to address this issue among Asian American women. The large sample size in this study allowed for analysis of most of the Asian ethnic subgroups surveyed in CHIS. CHIS 2001 was conducted in several languages allowing less acculturated and/or more recently immigrated Asians to be included in the analysis. The survey was also reviewed by experts to assure cultural compatibility and comprehensibility to target population groups. The demographic distributions of CHIS respondents were comparable to those from other surveys, suggesting that the survey was representative [38].

Our study also had several weaknesses. Because it was a secondary data analysis, we were limited in the variables we could examine. In particular, our dependent variable is a limited assessment of mammography use in the past two years. Because the information on screening and predictors were all based on self-report, differential misclassification may occur if misclassification in self-reporting of the predictor variables was associated with misclassification in reporting of mammography. Future studies designed specifically to address this question using RP might also incorporate cultural factors, such as knowledge and perceptions about preventive medical care, cancer screening, and breast cancer risk [26,51,52]; and modesty and embarrassment issues associated with mammography [27,52,53]. The response rates for CHIS 2001 were relatively low, yet these response rates are similar to the rates found in other population health surveys, such as the 2002 BRFSS [38]. Since CHIS is a telephone-based survey, selection biases such as non-coverage of households without telephones and households who "screen" telephone calls, as well as higher response rates among individuals of higher socioeconomic status, inherent to telephone surveys may exist.

## Conclusion

In addition to documented socioeconomic and financial barriers, our findings suggest institutional, cultural, and linguistic barriers for Asian-American women meeting mammography screening recommendations. Specifically, our results suggest that there are three major categories of Asian American women who require additional targeted efforts to improve their screening behaviors, and that different approaches are required for each group. The first group of women includes those who have health insurance and access their health care but do not see providers who specialize in women's health issues. This group may benefit from receiving reminders through their primary care providers or health care plan. The second group includes those who have health insurance and a usual source of care but who do not routinely access health care. These women tend to be more likely to be recent immigrants or non-citizens and have limited English facility. Public health and health-care based interventions should continue to focus on culturally- and linguistically-appropriate strategies for targeting these women. The third group includes those who do not have health insurance and/or a usual source of care. Outreach for this group could be effectively achieved through promoting screening through public insurance carriers, state-funded screening programs, and community-based interventions. Together with these methods, our data should be helpful in providing direction to those designing and implementing future interventions of benefit to the community of Asian women and in increasing rates of cancer screening in this group.

## Competing interests

The author(s) declare that they have no competing interests.

## Authors' contributions

SLG conceived of the study, conducted aspects of the data analysis, and drafted the manuscript. ST participated in the design of the study, conducted the data analysis, and drafted the manuscript. THMK and CAC participated in the interpretation of data and drafted the manuscript.

## Pre-publication history

The pre-publication history for this paper can be accessed here:


